# The role of Spanish clinical pharmacologists in economic evaluations of health technologies

**DOI:** 10.1007/s00228-026-04070-2

**Published:** 2026-05-07

**Authors:** Claudia Erika Delgado-Espinoza, Rosa M. Antonijoan, María Jose Martínez-Zapata

**Affiliations:** 1 Department of Pharmacology and Therapeutics, Universitat Autónoma de Barcelona (UAB), Barcelona, Spain; 2 Department of Clinical Pharmacology, Hospital de la Santa Creu i Sant Pau, Barcelona, Spain; 3 Institut de Recerca Sant Pau (IR SANTPAU), Barcelona, Spain; 4 Iberoamerican Cochrane Centre-Public Health and Clinical Epidemiology Service, IR Sant Pau, Barcelona, Spain; 5 Department of Paediatrics, Obstetrics, Gynaecology and Preventive Medicine and Public Health, Universitat Autónoma de Barcelona (UAB), Barcelona, Spain; 6 Centro de Investigación Biomédica en Red de Epidemiología y Salud Pública (CIBERESP), Madrid, Spain

**Keywords:** Clinical pharmacologists, Economic evaluations, Health technologies, Healthcare provider’s perspective, Spanish national health system

## Abstract

**Purpose:**

This study aimed to describe the role and perceptions of clinical pharmacologists (CPs) in conducting economic evaluations of health technologies within the Spanish National Health System from a healthcare provider perspective.

**Methods:**

We conducted a cross-sectional descriptive study using an online survey distributed to members of the Spanish Society of Clinical Pharmacology between September 2024 and September 2025. Eligible participants were CPs working within or linked to the Spanish National Health System. The questionnaire included four sections addressing respondent characteristics, direct involvement in economic evaluations, economic evaluations conducted by other professionals, and training and opinions. Data were analyzed using descriptive statistics.

**Results:**

Of 106 eligible CPs working within or linked to the Spanish National Health System, 48 completed the survey (response rate: 45.3%). The mean age was 51 years, and 56.3% were women. More than half of respondents (54.2%) reported conducting or having conducted economic evaluations, mainly cost-effectiveness and cost-minimisation analyses, often in collaboration with other healthcare professionals. Results were integrated into care protocols in 15 cases, although follow-up and outcome verification were performed in 7 cases. Among CPs not directly involved, 63.7% reported that economic evaluations are conducted at their centres by other professionals. Despite 91.7% of respondents considered that economic evaluation of health technologies is an activity that should be conducted in their centres, and all considered that a CP should participate in these evaluations, only 35.4% felt sufficiently trained, while 77.1% expressed interest in further training.

**Conclusion:**

Among the surveyed CPs, there is active involvement and a high level of motivation to participate in economic evaluations of health technologies in Spain. Strengthening training opportunities and collaborative networks could enhance their contribution to value-based healthcare.

**Supplementary Information:**

The online version contains supplementary material available at 10.1007/s00228-026-04070-2.

## Introduction

The continuous advancement of medicine in terms of new medications, medical devices, diagnostic methods, and other therapeutic interventions has led to improved health and quality of life for the population.

However, the implementation of new healthcare interventions requires demonstration of their efficacy in the context of clinical trials and their effectiveness in the context of routine clinical practice [1], as well as their safety.

Furthermore, considering that the resources allocated to public healthcare are limited [2] and that, therefore, every healthcare centre must be committed to the responsible and equitable use of these resources, it is important to demonstrate the efficiency of the interventions, understood as a process aimed at maximizing benefits, so that they can be reflected in improved health for the population we serve.

To achieve efficient use of available healthcare resources in an increasingly restrictive economic environment with growing healthcare demand, it is necessary to consider both health outcomes and costs [3].

According to Spanish Order SCO/3129/2006, which approves and publishes the training program for the specialty of Clinical Pharmacology in Spain [4], Clinical Pharmacology is the medical specialty that evaluates the effects of drugs in humans at the population level, and also in specific subgroups and individual patients. This evaluation focuses on the relationship between therapeutic effects (benefits), undesirable effects (risks), and the costs of therapeutic interventions, including efficacy, safety, effectiveness, and efficiency.

Clinical Pharmacology departments are involved in evaluating the efficiency of health technologies, it means medicinal products, medical devices or medical and surgical procedures as well as measures for disease prevention, diagnosis or treatment used in healthcare [5], with the aim of achieving the best possible application and optimizing available resources. In this sense, not only costs are taken into consideration, but also the cost-health outcome ratio.

For the economic evaluation of a healthcare intervention [6], a set of tools and procedures are designed to examine the potential consequences in terms of cost of implementing the intervention considering the resources used by the intervention and the consequences of its implementation.

We are immersed in a healthcare model in which aspects related to the assessment of the costs and benefits of decisions made in a context of growing needs and limited resources [7] are increasingly important. Given this scenario, economic evaluation studies serve as essential tools that support both healthcare professionals involved in patient care and those responsible for healthcare management in making informed decisions aimed at optimizing available resources, while consistently ensuring benefits for patients’ health and quality of life.

Based on these premises, this study aimed to describe the role and perceptions of clinical pharmacologists (CPs) in conducting economic evaluations of health technologies from the healthcare provider’s perspective as part of their profesional activities within or linked to centres across all levels of care in the Spanish National Health System.

## Methods

We conducted a cross-sectional descriptive study using an online survey, from September 2024 until September 2025. Inclusion criteria were to be a clinical pharmacologist (CPs) working within or linked to the Spanish National Health System.

To carry out the recruitment, we contacted the board of directors of the Spanish Society of Clinical Pharmacology (SEFC) asking for the distribution of the survey. The SEFC secretary sent the link to the survey via email to the society members’ distribution list, with three reminders throughout the study period. Participation in the survey was free and voluntary.

The survey was hosted on the Clinapsis platform (www.clinapsis.com) of the Sant Pau Research Institute, which has security measures in place to ensure compliance with data protection regulations.

The survey questionnaire included four sections with questions about (i) respondent characteristics, (ii) involvement of the respondents in economic evaluations of health technologies, (iii) activity on economic evaluations of health technologies conducted by other professionals, and (iv) training and opinions on the topic. The questionnaire comprised yes/no and multiple-choice questions, often accompanied by open-ended (free-text) options.

Data was analysed using descriptive statistics, using Microsoft Excel functions. Answers were summarised and examined through frequency tables. Answers to open-ended questions, given as comments in free-text boxes, were analysed in an individual manner. We calculate mean and standard deviations (SD) for continuous outcomes.

## Results

Email invitations to complete the survey were sent out to all 363 SEFC members. According to society records, 106 of these members were CPs working within or linked to the Spanish National Health System, constituting the eligible participants for this study.

A total of 50 invitees initiated the survey, and 48 of them responded and fully completed the survey, representing a 45.3% response rate.

### Respondents’ characteristics

Table 1 shows demographic characteristics, university and specialisation studies, and employment of the participants. Mean age was 51 years (SD 12.9). Of the respondents, 56.3% were women.

**Table 1:** Respondent characteristics

1. Age (mean (SD))	51 (12.9)
	*N* (%)
2. Gender	
Men	21 (43.7)
Women	27 (56.3)
3. Country where medical degree was obtained	
Spain	38 (79.2)
Europe (excluding Spain)	2 (4.2)
Latin American country	8 (16.6)
4. Country where specialty training in Clinical Pharmacology was completed	
Spain	47 (97.9)
Latin American country	1 (2.1)
4.1. Years of practice as a Clinical Pharmacologist	
Less than 5 years	11 (22.9)
5–10 years	4 (8.3)
> 10 years	33 (68.8)
5. Primary workplace	
Hospital	36 (75.0)
Primary care	3 (6.3)
Others*: research institute/university	9 (18.8)
5.1. Current professional position	
Researcher	4 (8.3)
Attending physician	22 (45.8)
Unit Manager/Clinical Director	7 (14.6)
Head of Department	9 (18.8)
Others*: consultant, coordinator, manager, professor	6 (12.5)

* “Others” include free text responses

As far as university medicine education and specialisation in clinical pharmacology, 79.2% reported that they studied at a Spanish university, and 97.9% obtained CP specialty in Spain, with a time of exercise of more than 10 years in the 68.8% of respondents.

Regarding primary workplace, 75% of respondents work in a hospital setting, 45.8% as attending physicians. A relevant proportion (18.8%) reported working in other settings, mainly research institutes or universities. In terms of professional position, respondents classified under “other” roles (12.5%) included consultants, coordinators, managers, and professors.

### Involvement of CPs in economic evaluations

Table 2 shows answers related to the involvement of participants in economic evaluations.

**Table 2:** Involvement in economic evaluations

	*N* = 48
	*n* (%)
6. As part of your activities as a clinical pharmacologist in a National Health System centre, have you conducted or are you currently conducting economic evaluations of health technologies?	
Yes	26 (54.2)
No	21 (43.7)
No response	1 (2.1)
7. Which other professionals have worked or are currently working with you on these economic evaluations? *	
Clinical Pharmacologists	18
Other** Healthcare Professionals: pharmacists, clinicians, epidemiologists	23
Other** Non-Healthcare Professionals: economists, statisticians	6
None	0
8. What type(s) of economic evaluation have you conducted or are you currently conducting? ***	
Cost-minimisation analysis	13
Cost-effectiveness analysis	14
Cost-utility analysis	4
Cost-benefit analysis	3
Others **: viability analysis, cost-consequence analysis, budget impact analysis, annual expenses analysis	5
9. In what context were or are the economic evaluations conducted? *	
Within a research project	14
As part of Clinical Pharmacology Department activities	12
As part of another department or functional unit activities	13
10. If the economic evaluation was conducted within a research project, what was the study design? *	
Clinical trial	10
Observational study	11
Others **: quasi-experimental, pre-post design	6
11. Who initiated the economic evaluations you conducted or are conducting? *	
Clinical Pharmacology Department initiative	13
Request from the Medical Director or Manager	11
Request from another Department	11
12. If you have conducted economic evaluations, have the results been integrated into your centre’s care protocols?	
Yes	15
No	7
I do not know	3
Results pending	1

* Multiple responses allowed

** “Other/s” include free text responses

*** Respondents could report involvement in more than one type of economic evaluation

It was found that 54.2% of respondents participate or have participated in economic evaluations, typically in association with other healthcare professionals, like pharmacists, clinicians, epidemiologists (*n* = 23) and CPs (*n* = 18); and to a lesser extent with non-healthcare professionals like economists and statisticians (*n* = 6).

The evaluations mainly involved cost-effectiveness (*n* = 14) and cost-minimisation analyses (*n* = 13). In addition to these standard approaches, a subset of respondents reported conducting other types of evaluations (*n* = 5), including viability analyses, cost-consequence analyses, budget impact analyses, and annual expense analyses.

These economic evaluations were performed in the context of research projects (*n* = 14), as part of another department or functional unit activities (*n* = 13), or as part of clinical pharmacology department activities (*n* = 12), in a similar number. The differentiation between these last two options allows for an assessment of whether economic evaluation is a primary responsibility of the Clinical Pharmacology departments or remains a collaborative support role for other clinical departments or multidisciplinary committees.

CPs who conducted economic evaluations as part of a research project, were mainly within observational studies (*n* = 11) or clinical trials (*n* = 10). Additionally, other study designs were described (*n* = 6), including quasi-experimental and pre–post designs.

Regarding the initiative for conducting economic evaluations, answers were homogeneous between the initiative of the clinical pharmacology department (*n* = 13), the request of the medical direction or manager (*n* = 11) or the request of another department (*n* = 11).

**Fig. 1:**
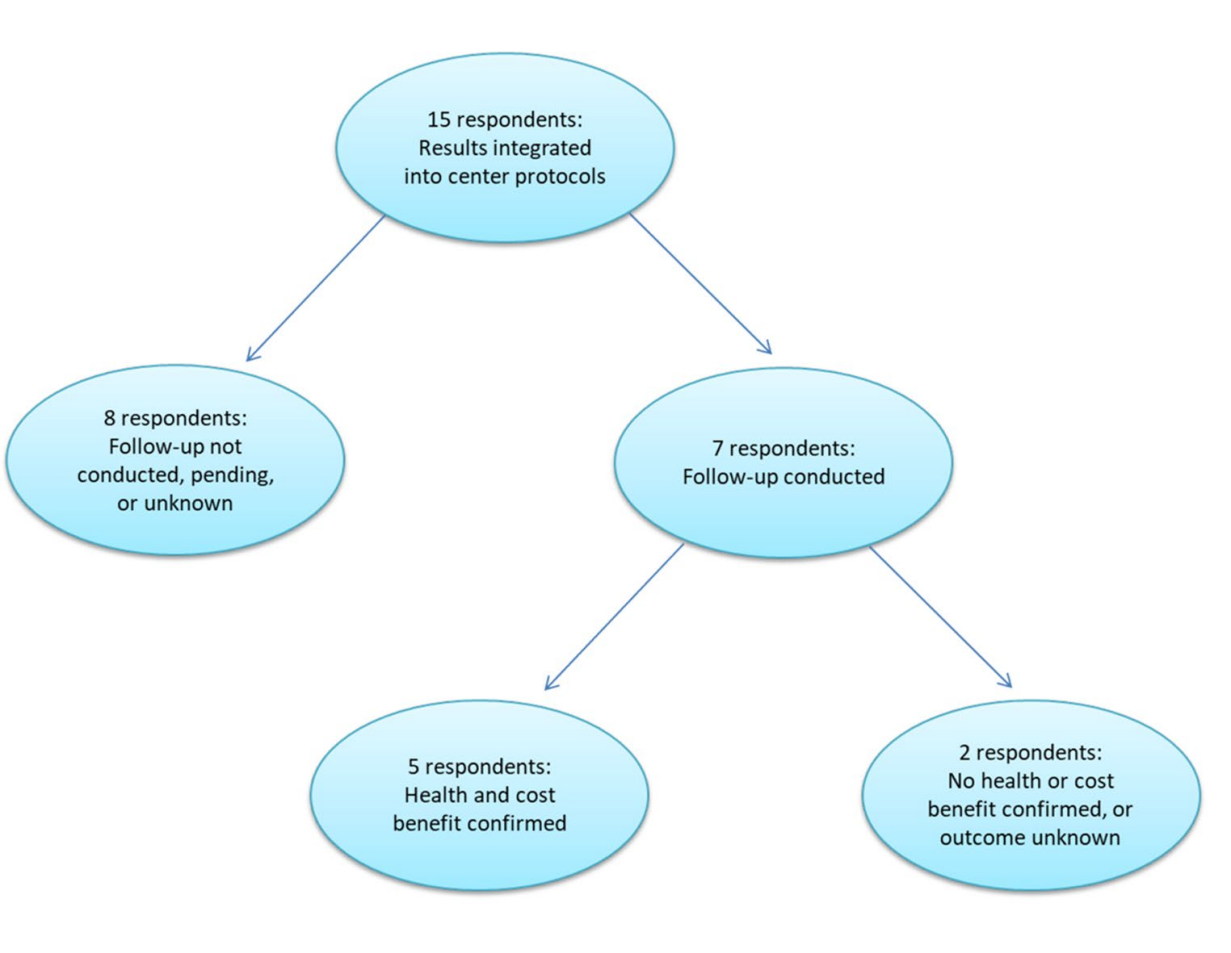
Integration, follow-up, and impact of economic evaluation results in centre care protocols

Of the CPs who conducted economic evaluations, 15 affirmed that the results were integrated in their centres’ care protocols. Of them, 7 affirmed that a follow-up was conducted to verify whether a health/cost benefit has been achieved, and 5 confirmed that a health/cost benefit was obtained (Fig. 1).

### Economic evaluations conducted by other professionals

Table 3 shows the answers to the questions related to the involvement of other professionals (not the respondents themselves) in economic evaluations of health technologies. This question was included to determine whether economic evaluation activities were conducted at respondents’ centres by other professionals, including both healthcare and non-healthcare professionals, as well as other CPs who may not have responded to the survey.

For this part of the survey, respondents were the 22 who responded not to conduct economic evaluations of health technologies by themselves.

Of them, 14 (63.7%) stated that in their centres, economic evaluations are or were conducted by other professionals, including healthcare professionals other than CPs (*n* = 8) and non-healthcare professionals (*n* = 3) such economists. Only 4 respondents stated that, in their centres, economic evaluations are or were conducted by other CPs.

**Table 3:** Activity on economic evaluations by other professionals

	*n* = 22
	*n* (%)
15. Are economic evaluation activities conducted at your centre, but conducted by professionals other than yourself?	
Yes	14 (63.7)
No	5 (22.7)
I do not know	3 (13.6)
15.1. If yes, which professionals conduct these economic evaluations? *	
Other clinical pharmacologists (CPs)	4
Other healthcare professionals non-CPs	8
Non-healthcare professionals	3
I do not know	1

* Multiple responses allowed

### Opinions on the topic and training

Table 4 shows the results regarding opinions on the topic and training.

**Table 4:** Opinions on the topic and training

	*N* = 48
	*n* (%)
16. Do you think that economic evaluation of health technologies should be conducted in your centre?	
Yes	44 (91.7)
No	4 (8.3)
17. Do you think that clinical pharmacologists should participate in the economic evaluation of health technologies?	
Yes	48 (100)
No	0 (0)
18. Would you be interested in pursuing economic evaluation of health technologies as part of your professional activity?	
Yes	33 (68.7)
No	7 (14.6)
I do not know	8 (16.7)
19. Do you feel sufficiently trained to conduct economic evaluations of health technologies?	
Yes	17 (35.4)
No	25 (52.1)
I do not know	6 (12.5)
20. Would you be interested in receiving training in economic evaluation of health technologies?	
Yes	37 (77.1)
No	3 (6.2)
I do not know	8 (16.7)

Most of the respondents (91.7%) felt that economic evaluation of health technologies is an activity that should be carried out in their centres, and all respondents considered that a CP should participate in these evaluations.

While 68.7% of respondents expressed interest in participating in economic evaluations of health technologies, 52.1% felt not sufficiently trained for such tasks and 77.1% wished to improve their knowledge.

## Discussion

This study reports the results of a national online survey designed to describe the role of CPs in conducting economic evaluations of health technologies from the healthcare provider’s perspective, as part of their routine professional activities within or linked to the Spanish National Health System. To our knowledge, this is the first survey conducted in Spain specifically addressing this topic, providing novel insight into both current practice and perceived needs within the specialty.

Previous surveys exploring economic evaluations have primarily focused on health economic researchers or decision-makers [8–11]. These investigations prioritized academic qualifications and institutional affiliations in regions such as the Gulf Cooperation Council [8] or low- and middle-income countries [9], but lacked detailed data on professional clinical profiles or medical specialisation [10, 11]. Compared with these studies, the present study focuses on clinical pharmacologists to evaluate their level of involvement in economic evaluations of health technologies.

Physician surveys are known to be an important tool in health services and policy research, although they are often characterized by low response rates [12]. In our study, the response rate was 45.3%, which is comparatively higher than those reported in surveys referenced previously [8, 9, 11], where response rates ranged from 19% to 37%. Published data [13] indicate that physician surveys in biomedical research typically achieve a mean response rate of approximately 54%, while response rates for email-based surveys in other contexts are often considerably lower [14]. Although the lack of demographic data for non-respondents precludes a formal demonstration of representativeness, the participation of nearly half of the eligible participants provides a reasonable overview of the target population.

The characteristics of the respondents reflect a mature professional group with a slight predominance of women, consistent with the increasing trend toward feminisation of the medical workforce in Spain, as reported by national statistics [15]. Importantly, almost one-third of respondents occupied leadership positions, either as heads of service or unit managers/clinical directors, while nearly half were attending physicians. This distribution supports the relevance of the findings for both clinical practice and organisational decision-making. As expected, hospitals constituted the main workplace for CPs, reflecting the relevance of the specialty within hospital-based care.

The fact that just over half of the respondents currently conduct economic evaluations of health technologies suggests that, while this practice is an important tool for healthcare managers in their strategies for allocating healthcare resources and adopting new technologies [16], CPs are not participating in these activities in a standardised way. Consequently, there is a clear need to promote these evaluations and to integrate CPs into multidisciplinary teams to conduct them.

Overall, these findings suggest a coherent but still limited integration of economic evaluation of health technologies within clinical pharmacology practice in Spain. CPs are involved, motivated, and aware of the strategic importance of economic evaluation of health technologies, yet variability persists in implementation, follow-up, leadership, and training.

These results are useful in identifying specific areas where the role of CPs in economic evaluations can be strengthened, providing a strategic roadmap for the national society of this specialty. Furthermore, these data offer healthcare decision-makers an overview of how economic assessments are currently integrated into clinical and research activities, which supports the wider implementation of such activities across the Spanish National Health System. Enhancing this professional involvement facilitates more robust resource management and evidence-based decision-making, ensuring that health benefits are maximised without compromising system sustainability.

The study also has implications for the future implementation of European Union (EU) Regulation 2021/2282 on health technology assessment (HTA) [5]. By identifying current levels of involvement, collaboration patterns, and training gaps, the findings highlight factors that should be reinforced to enable CPs to play a strategic role in conducting HTA processes at the EU level.

Key strengths of the study include its novelty, as to our knowledge, it is the first to specifically examine the involvement of CPs in economic evaluations of health technologies in the context of the Spanish National Health System. Furthermore, the relative high response rate provides a comprehensive national overview of this topic, offering valuable baseline data for enhance professional opportunities and promote institutional recognition of CPs in the field of health economics.

The study is firstly limited by its small absolute sample size, Additionally, eligible participants identified through the SEFC may not constitute the entire universe of CPs involved in economic evaluations of health technologies in centres of the Spanish National Health System. Consequently, although the SEFC likely encompasses the majority of specialists in the field, the results should be interpreted with caution and may not be fully generalisable to CPs who are not members of the society or those working in different healthcare settings.

Secondly, the retrospective nature of the survey may have introduced recall bias, as respondents might not have recalled every specific detail of evaluations conducted throughout their professional careers, nor the overlap between research and routine clinical activities inherent to the clinical pharmacology specialty. Nevertheless, the global nature of some questions was designed to avoid excessive respondent burden and ensure a high completion rate, providing a broader overview that reflects CPs activities in economic evaluations.

Finally, it must be considered that the findings are context-specific to the Spanish National Health System, which may restrict their applicability to other international settings.

In conclusion, among the surveyed CPs, there is active involvement and a high level of motivation to participate in economic evaluations of health technologies in Spain but there remains substantial room for increased participation and enhanced training opportunities. Future research should extend this work at a European level and explore professional networks and collaborative practices to support harmonized training, cross-border collaboration, and a stronger multidisciplinary community focused on efficient and sustainable healthcare.

## Supplementary Information

Below is the link to the electronic supplementary material.

Supplementary Material 1

## Data Availability

The datasets of the current study are available from the corresponding author on reasonable request.
