# Supplementary material for: The role of Spanish clinical pharmacologists in economic evaluations of health technologies
Source: Eur J Clin Pharmacol. 2026 May 7;82(6):141. doi: 10.1007/s00228-026-04070-2 (PMC13149689; doi:10.1007/s00228-026-04070-2)
Supplement: Supplementary file 1 — Supplementary Material 1 [file 228_2026_4070_MOESM1_ESM.docx]

**SUPPLEMENTARY MATERIAL: SURVEY**

**GENERAL PART:**

* “Other/s” include free text responses.

1. Age

2. Gender

3. Which university did you study at?

4. Where did you obtain your specialty in Clinical Pharmacology?

4.1. In what year did you obtain your specialty in Clinical Pharmacology?

5. Where do you work?

- Hospital
- Primary care
- Others (specify) *

5.1. What is your job position?

- Researcher
- Attending physician
- Unit Manager/Clinical Director
- Head of Department
- Others (specify) *

**ECONOMIC EVALUATIONS:**

* Multiple responses allowed.

** “Other/s” include free text responses.

*** Respondents could report involvement in more than one type of economic evaluation.

6. As part of your activities as a clinical pharmacologist in a National Health System centre, have you conducted or are you currently conducting economic evaluations of health technologies?

- Yes
- No

7. Which other professionals have worked or are currently working with you on these economic evaluations? *

- Clinical Pharmacologists
- Other Healthcare Professionals
- Other Non-Healthcare Professionals (specify) **
- None

8. What type(s) of economic evaluation have you conducted or are you currently conducting? ***

- Cost-minimisation analysis
- Cost-effectiveness analysis
- Cost-utility analysis
- Cost-benefit analysis
- Others (specify) **

9. In what context were or are the economic evaluations conducted? *

- Within a research project
- As part of Clinical Pharmacology Department activities
- As part of another department or functional unit activities

10. If the economic evaluation was conducted within a research project, what was the study design? *

- Clinical trial
- Observational study
- Others (specify) **

11. Who initiated the economic evaluations you conducted or are conducting? *

- Clinical Pharmacology Department initiative
- Request from the Medical Director or Manager
- Request from another Department

12. If you have conducted economic evaluations, have the results been integrated into your centre's care protocols?

- Yes
- No
- I do not know
- Results pending

13. If the results of economic evaluations have been integrated into your center's care protocols, has follow-up been conducted to verify whether a health/cost benefit has been achieved?

- Yes
- No
- I don't know
- Pending follow-up

14. If compliance with healthcare protocols has been monitored in relation to recommendations related to economic evaluations, has it been confirmed whether a health/cost benefit has occurred?

- Yes
- No
- I don't know

**ECONOMIC EVALUATIONS CONDUCTED BY OTHER PROFESSIONALS**

* Multiple responses allowed.

15. Are economic evaluation activities conducted at your centre, but conducted by professionals other than yourself?

- Yes
- No
- I do not know

15.1. If yes, which professionals conduct these economic evaluations? *

- Other clinical pharmacologists (CPs)
- Other healthcare professionals non-CPs
- Non-healthcare professionals
- I do not know

**OPINIONS ON THE TOPIC AND TRAINING**

16. Do you think that economic evaluation of health technologies should be conducted in your centre?

- Yes
- No

17. Do you think that clinical pharmacologists should participate in the economic evaluation of health technologies?

- Yes
- No

18. Would you be interested in pursuing economic evaluation of health technologies as part of your professional activity?

- Yes
- No
- I do not know

19. Do you feel sufficiently trained to conduct economic evaluations of health technologies?

- Yes
- No
- I do not know

20. Would you be interested in receiving training in economic evaluation of health technologies?

- Yes
- No
- I do not know
